# The metagenome and metabolome signatures of dental biofilms associated with severe dental fluorosis

**DOI:** 10.1080/20002297.2025.2560591

**Published:** 2025-09-23

**Authors:** Penpitcha Ajrithirong, Annop Krasaesin, Wannakorn Sriarj, Patcharaporn Gavila, Wanna Chetruengchai, Kanokwan Sriwattanapong, Chawan Manaspon, Lakshman Samaranayake, Thantrira Porntaveetus

**Affiliations:** a Department of Pediatric Dentistry, Faculty of Dentistry, Chulalongkorn University, Bangkok, Thailand; b Department of Physiology, Faculty of Dentistry, Center of Excellence in Precision Medicine and Digital Health, Chulalongkorn University Implant and Esthetic Center, Chulalongkorn University, Bangkok, Thailand; c Program in Bioinformatics and Computational Biology, Graduate School, Chulalongkorn University, Bangkok, Thailand; d Intercountry Centre for Oral Health, Department of Health, Ministry of Public Health, Chiangmai, Thailand; e Center of Excellence for Medical Genomics, Department of Pediatrics, Faculty of Medicine, Chulalongkorn University, Bangkok, Thailand; f Excellence Center for Genomics and Precision Medicine, King Chulalongkorn Memorial Hospital, the Thai Red Cross Society, Bangkok, Thailand; g Biomedical Engineering Institute, Biomedical Engineering and Innovation Research Center, Chiang Mai University, Chiang Mai, Thailand; h Faculty of Dentistry, University of Hong Kong, Hong Kong; i Global Research Cell, Dr. D. Y. Patil Dental College and Hospital, Dr. D. Y. Patil Vidyapeeth, Pimpri, Pune, India; j Clinic of General-, Special Care and Geriatric Dentistry, Center for Dental Medicine, University of Zurich, Zurich, Switzerland

**Keywords:** Oral microbiome, health disparity, caries, bacteria, esterase, symbiosis, dental plaque, skeleton, remineralization

## Abstract

**Objective:**

To explore the plaque biofilm microbiome associated with severe dental fluorosis (SF), and to describe its metagenome and metabolome.

**Methods:**

Sixteen plaque biofilm samples were collected from eight 6- to 15-year-old Thai children with SF and eight age-matched, caries-free and controls. Biofilms were analyzed using shotgun metagenomic sequencing, followed by bioinformatics evaluation.

**Results:**

Taxonomic profiling of biofilms from SF and controls identified a total of 12 phyla and 354 species. While alpha diversity was similar between the groups, beta diversity analysis (*P* = 0.0010) indicated distinct microbial community structures. LEfSe highlighted key discriminatory taxa: five health-associated species (*Actinomyces dentalis, Tannerella sp. HOT 286, Candidatus Nanosynbacter sp, Selenomonas noxia* and *Treponema sp OMZ 804* ) were enriched in controls, while *Neisseria sicca*, known for fluoride-sensitive esterase production, was significantly elevated in SF. Functionally, eight metabolic pathways were altered; three of these (phosphatidylcholine acyl editing, anhydromuropeptides recycling II, ubiquinol-7 biosynthesis), hypothesized to support *N. sicca* activity, were upregulated in the SF group.

**Conclusion:**

SF is associated with a significant shift in the biofilm microbiota, characterized by enrichment of *N. sicca* and a reduction in health-associated taxa. Altered metabolic pathways supporting *N. sicca* provide mechanistic insights into its role as a candidate biomarker for fluorosis, warranting further investigation.

## Introduction

Community water fluoridation has been a long-standing a public health measure to prevent dental caries. Despite its widespread adoption and endorsement by major health organizations for safety and effectiveness at recommended levels, it remains a contentious topic of ethical and scientific debate [1]. Critics argue that fluoridation amounts to mass medication without individual consent, potentially infringing on personal autonomy. Furthermore, recent studies have raised concerns about possible links between fluoride exposure and adverse neurodevelopmental or systemic health effects [2]. With increasing access to fluoride from alternative sources – including toothpaste, mouth rinses, milk and salt – questions about the necessity and safety of universal water fluoridation have emerged, particularly in relation to the risk of overexposure in specific populations. These ongoing concerns underscore the need for a thorough evaluation of fluoride exposure and its potential health impacts.

Dental fluorosis (DF), is a relatively common oral health issue, resulting from excessive fluoride intake during tooth development [3]. While fluoride is essential for preventing dental caries, its overexposure can lead to enamel hypomineralization, leading to esthetic and functional problems. These include horizontal white lines, opacities, discoloration (mottling), pitting, and in severe cases, enamel collapse, causing tooth sensitivity, mastication difficulties and even skeletal fluorosis [4,5]. The severity of DF is influenced by the fluoride level and exposure duration [6].

Globally, an estimated 200 million people are exposed to fluoride concentrations exceeding the World Health Organization (WHO)'s recommended limit of 1.5 mg/L in drinking water [7]. In regions such as Ratchaburi and Chiang Mai provinces in Thailand, natural sources have led to fluoride levels in groundwater surpassing the recommended limit resulting in widespread DF of the populace [8].

Recent advancements in microbiome research have underscored the critical role of microbial communities in maintaining human health [9]. The oral cavity harbors a diverse microbiome, comprising approximately 700 taxa, which exists in a complex eubiotic equilibrium with the host [10]. Disruptions to this equilibrium, known as dysbiosis [11–13], have been linked to oral diseases, including dental caries, periodontal disease and halitosis [10]. Emerging evidence also indicate that a dysbiotic oral microbiome may also be associated with DF [1]. Consequently, distinct microbial signatures or shifts in microbial populations could serve as potential biomarkers, reflecting the physiological impact of fluoride on the oral ecosystem and potentially aiding in the characterization or early detection of fluorosis. However, there is no data on the microbiome of severe dental fluorosis (SF) in comparison to a healthy eubiotic microbiome.

Hence, we investigated the dental biofilm microbial composition and its metabolome in SF versus healthy controls, hypothesizing that SF induces a distinct oral microbial signature. Using comprehensive shotgun metagenomics, we aimed to elucidate the role of microbiome in DF, potentially informing future clinical management strategies.

## Materials and methods

### Study design and subject recruitment

The research protocol was approved by the Human Research Ethics Committee of the Faculty of Dentistry, Chulalongkorn University, Bangkok, Thailand (HREC-DCU 2021-031). A total of 214 students from Ratchaburi and 39 from Chiang Mai, aged 6−15, were recruited through a systematic approach during December 2021 and January 2022. Schools and community health centers in the target provinces helped identify potential participants. Information sessions were held to explain the study objectives, procedures, and benefits to both parents and children, after which informed consent was obtained from both cohorts.

Data were recorded using a form based on the recommendations and nomenclature of the WHO Oral Health Surveys [14]. Individuals with clinical dental caries, periodontitis, oral mucosal lesions, other systemic diseases, and current smoking, alcohol use or drug abuse, had taken antibiotics, antiviral drugs, or steroids, used mouthwashes within the last 3 months, undergone professional teeth cleaning within the last 30 days, or received any dental treatment for teeth affected by fluorosis were excluded from the study.

**Figure 1.**
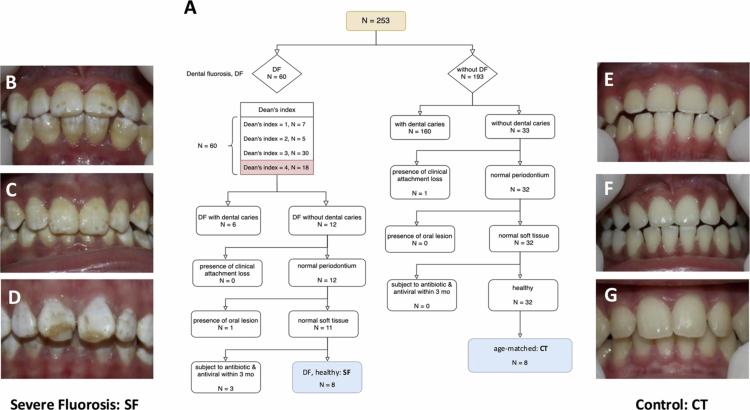
Flow chart of the study design and clinical features of participants in this study. (A) The flow chart of study design in this study. (B–D) The clinical features of the SF group defined with discrete or confluent pitting, widespread brown stains which present as a corroded-like appearance whereas the control group (E–G) revealed shine smooth, glossy with translucent creamy-white color of the normal enamel. CT = control, SF = severe fluorosis.

The inclusion criteria for the SF group in this study consisted of individuals from the Thai population exhibiting severe dental fluorosis, classified as 'SF' based on the Dean's Index score of 4. A consensus score for Dean’s Index was provided by two experienced dentists (P.A. and P.G.), with the final score recorded directly in an Excel file. Inter-observer reliability for the Dean’s Index was assessed by having both examiners independently evaluate all participants. Intra-observer reliability was confirmed by both examiners re-evaluating all participants after a minimum interval of three weeks. The Kappa coefficients (0.85 for inter-examiner reliability, 0.90 for intra-examiner-1, and 0.87 for intra-examiner-2), indicated excellent agreement between examiners. Participants without DF served as a control group. The study included 8 patients with SF and 8 healthy age-matched controls (Figure 1). This balanced design was employed to enhance the reliability of ANOSIM statistics. Although all participants were recruited from the same geographic area, none were from the same household.

### Collection and fluoride determination in water samples

The participants were asked to collect the most consumed drinking water in their household. The water samples were collected in 60 ml clean bottles, approximately 5/6 full. The samples were kept at room temperature and transported to the laboratory at the Intercountry Center for Oral Health in Chiang Mai, Thailand. The fluoride concentration was evaluated using a fluoride electrode (4-star benchtop, Orion, USA) as the measuring equipment and TISAB III, containing cyclohexylenedinitrilotetraacetate (CDTA), sodium hydroxide, sodium chloride and acetic acid (ethanoic acid) dissolved in deionized water.

### Plaque biofilm collection and shotgun metagenomic sequencing

Participants were instructed not to eat or drink after midnight prior to sample collection, and not to brush their teeth the night before or the morning prior to plaque collection. Supragingival biofilm was collected from every tooth in the oral cavity by a single examiner (PA) using a sterile explorer and pooled into 1.5 ml tubes. After collection, the samples were placed in a foam container with dry ice, and immediately transported to the laboratory, and stored at –80 °C for further analysis.

DNA was extracted from the samples using the QIAamp DNA Microbiome Kit (Qiagen, Hilden, Germany) following the manufacturer’s protocols. The samples were then subjected to shotgun metagenomic sequencing using the TruSeq Nano DNA Library Prep Kit and the Illumina Novaseq system with 2 × 151 bp read lengths (Macrogen, Seoul, South Korea) utilizing sequencing by synthesis technology. A total of 205.7 GB of raw sequencing data in FASTQ format was generated from 16 samples.

### Metagenomic analysis

Quality control screening and removal of host contamination from raw reads were performed using KneadData version 0.12 (http://bitbucket.org/biobakery/kneaddata), which integrates Trimmomatic version 0.33 and Bowtie2 version 2.5.1. Low-quality sequences were filtered out and mapped to the human genome (hg19) using default parameters to remove host contamination prior to further analysis.

Taxonomic assignment and abundance estimation were conducted using MetaPhlAn (v4.0) [15] with default parameters. Microbial profiles, including full taxonomy, were classified into different levels – kingdom, phylum, class, order, family, genus and species – using an in-house script. Alpha diversity of species level, measured by Shannon, Simpson and Chao1 indices to assess species diversity within each group, was evaluated and visualized using Scikit-Bio library version 0.6.0, Matplotlib version 3.8.4, and Seaborn version 0.13.2 libraries, in Python version 3.11.4. Differences in alpha diversity between SF group and control group were tested using the Wilcoxon rank sum test.

Beta diversity was assessed using Bray–Curtis metrics to examine dissimilarities in bacterial communities across groups. The corresponding distance matrices and principal coordinate analysis (PCoA) were also conducted and visualized in Scipy library version 1.13.0, Matplotlib version 3.8.4, and Seaborn version 0.13.2 libraries, in Python version 3.11.4. Permutational multivariate analysis of variance (PERMANOVA) was performed to compare microbial composition differences between groups. A *p*-value < 0.05 was considered statistically significant. Differences in the relative abundance of microbial taxa between SF and control groups were visualized as a heatmap with log normalization. Biomarkers were identified using linear discriminant analysis effect size (LEfSe) with an LDA score above 2 and a *p*-value of < 0.05.

Metabolic profiles were performed and estimated by HUMAnN (v3.6) [16]. The functional profiles as related pathways were selected by significant differences with the False Discovery Rate (FDR) using Benjamini–Hochberg methods, statistically significant at FDR < 0.05 by SciPy library version 1.13.0 in Python version 3.11.4. A heatmap of significant pathways was conducted to observe the cluster in each group by scaling data with *Z*-score normalization. Statistical FDR and fold change differences determined the highlighted pathways as volcano plots and log2 fold change (log2FC) bar plots. All illustrations were created using Matplotlib version 3.8.4 and Seaborn version 0.13.2 libraries in Python version 3.11.4.

## Results

### Subjects and metagenomics data

Among the 253 surveyed students, the prevalence of DF was 23.7% (*N* = 60). Eighteen had a Dean’s Index score of 4. Only eight in this group were eligible for the metagenomics study as, six had active caries, one had a dentoalveolar abscess and 3 had received antibiotic or antiviral treatment in the past 3 months (Figure 1A–D). The same criteria were used to recruit the control group, resulting in eight eligible controls (Figure 1E–G). The mean age of the SF group was 10.38 ± 2.62 years, with 4 males and 4 females, while the mean age of the control group was 10.75 ± 2.55 years, with 6 males and 2 females. The mean fluoride levels in water for the SF group were 1.64 ± 2.63 mg/L (range 0.14–5.9), compared to 0.82 ± 1.93 mg/L (range 0–5.6) for controls.

A total of 1,363,617,198 paired-end reads were generated using the Illumina Novaseq platform, with an average of 85,226,074.88 reads per sample, ranging from 66,756,738 to 114,489,216. After filtering and removing artificial sequences, 1,284,948,648 high-quality reads were retained, with a mean of 80,309,290.5 sequences per sample and a range of 63,072,726–105,848,934 sequences. To assess sequencing depth, we performed rarefaction analysis using RPK values from the HUMAnN3 pipeline. As shown in Supplementary Figure S1, the curves plateau, indicating sufficient sequencing depth to capture most functional gene diversity in the samples.

The analysis of 16 test and control plaque biofilm samples using the NGS technology revealed 12 abundant phyla comprising 45 classes, 58 orders, 73 families, 138 genera and 354 species.

### Microbial composition

In the SF group, *Proteobacteria* was the most abundant phylum (39.70 ± 7.70%), followed by *Firmicutes* (20.32 ± 9.48%) and *Actinobacteria* (15.83 ± 11.66%). The most frequent genera were *Neisseria* (22.00 ± 4.76%), *Streptococcus* (10.53 ± 6.28%) and *Actinomyces* (7.11 ± 4.83%). At the species level, *Neisseria sicca* (13.47 ± 5.38%) was the most abundant, followed by *Lautropia mirabilli* (5.80 ± 3.87%), *Streptococcus sanguinis* (4.92 ± 2.27%), *Haemophilus parainfluenzae* (4.54 ± 3.06%) and *Neisseria elongata* (4.13 ± 3.15%).

**Figure 2.**
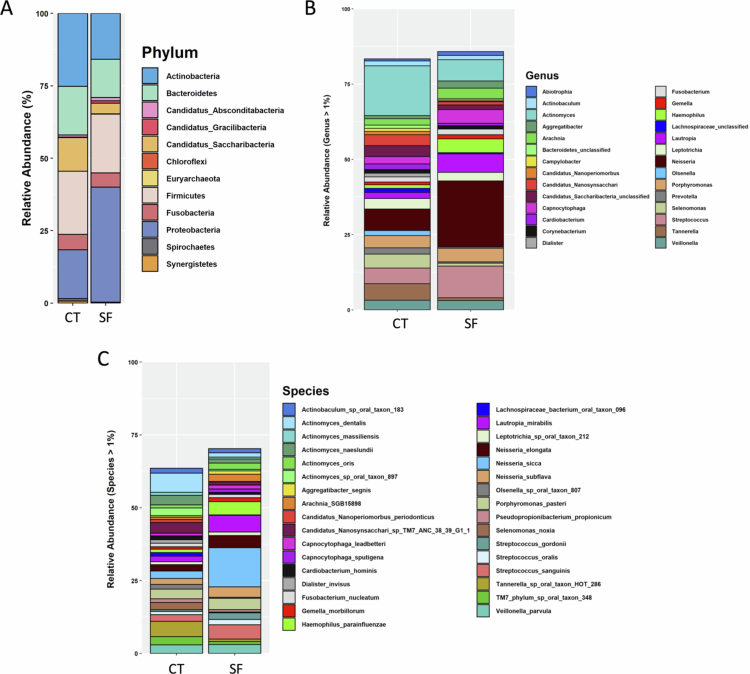
Bar graph of average relative abundance in different taxonomic levels found in both severe dental fluorosis (SF) and control (CT) group. (A) The average proportion of relative abundance at the phylum level which showed that *Proteobacteria* (39.70 ± 7.70%) was the most abundant phyla found in SF group while *Actinobacteria* (25.19 ± 11.78%) was the most frequent phyla found in the control group. (B) The most abundant genus in SF group was *Neiserria* (22.00 ± 4.76%) whereas *Actinomyces* (16.51 ± 7.27%) was the most frequent genus found in the controls. (C) At species levels, *Neiserria sicca* (13.47 ± 5.38%) was the most abundant in SF group and *Actinomyces dentalis* (6.60 ± 3.65%) was the most frequent species in the control.

In the control group, *Actinobacteria* (25.19 ± 11.78%) was the most abundant phylum, followed by *Firmicutes* (21.78 ± 7.99%), and *Proteobacteria* (16.82 ± 6.57%). The most frequent genera were *Actinomyces* (16.51 ± 7.27%), *Neisseria* (7.15 ± 43.91%) and *Tannerella* (5.45 ± 3.77%). At the species level, *Actinomyces dentalis* (6.60 ± 3.65%), *Tannerella sp. oral taxon HOT 286* (5.23 ± 3.70%), *Candidatus nanosynsacchari sp. TM7 ANC 38.39 G1 1* (3.64 ± 2.04%), *Porphyromonas pasteri* (3.37 ± 2.96%) and *Actinomyces naeslundii* (3.25 ± 2.49%) were the most abundant (Figure 2A–C, Supplementary Table S1).

### Microbial diversity and abundance

**Figure 3.**
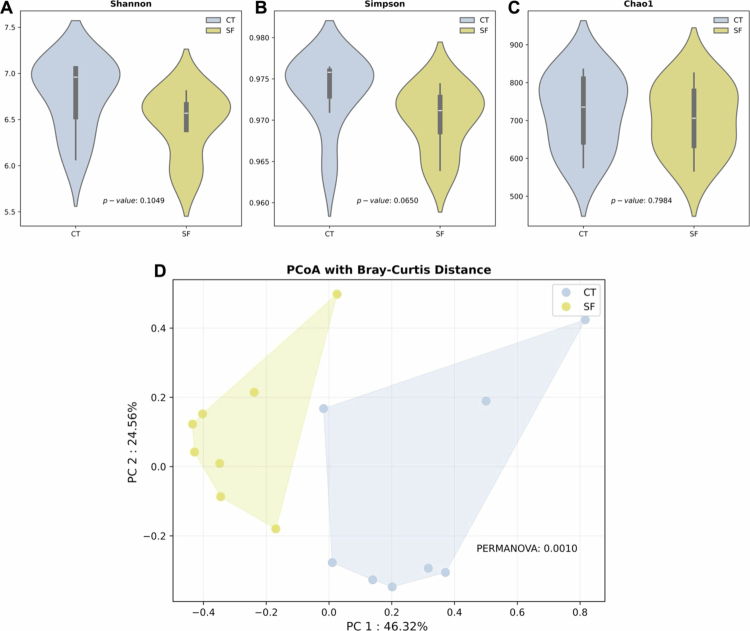
Violin plot of alpha diversity indices and principal coordinates analysis (PCoA) with Bray–Curtis. (A–C) The alpha diversity based on Shannon, Simpson and Choa1 showed no significant differences between groups (*p*-value = 0.0149, 0.0650, and 0.7964, respectively). (D) The beta diversity, the PCoA plot based on Bray–Curtis showed a significant divergence between the SF group and control group (PERMANOVA, *p*-value = 0.0010). CT = control, SF = severe fluorosis.

Alpha diversity, measured by the Shannon, Simpson and Chao1 indices using species level, showed no significant difference between the SF and control groups (Figure 3A–C). Bray–Curtis PCoA metrics used to evaluate the beta diversity of the biofilm microbiome between the SF and controls, revealed significant and distinct clusters (PERMANOVA, *p* = 0.0010) (Figure 3D).

**Figure 4.**
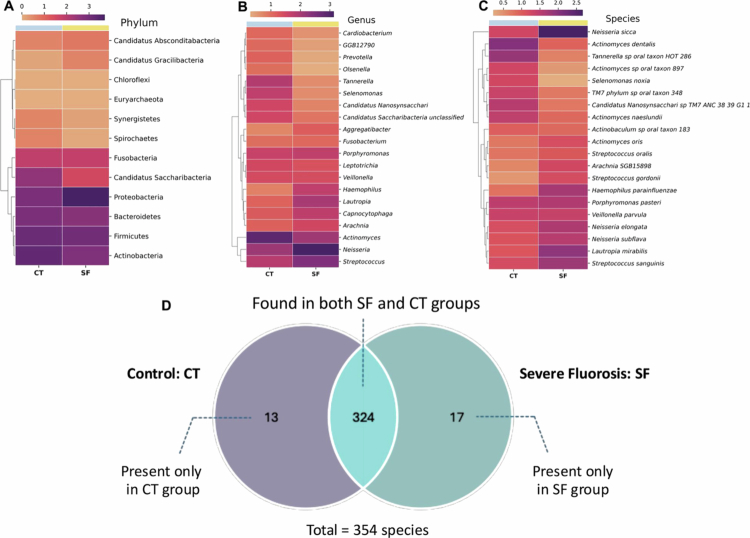
Heatmaps at each taxonomic level and a Venn diagram illustrating species distribution in the severe dental fluorosis (SF) and control (CT) groups. (A–C) Heatmap analysis illustrates the 20 most abundant microbial taxa in dental biofilms, comparing the SF and CT groups. Data are presented at the phylum, genus and species levels, with color intensity corresponding to relative abundance. (D) The Venn diagram shows the overlap and uniqueness of species detected in both groups. A total of 324 species were shared between SF and CT groups, while 17 species were exclusively found in the SF group and 13 species were unique to the CT group. CT = control, SF = severe fluorosis.

The heatmap revealed the top 20 microbial taxa, ranked by relative abundance, in dental biofilms between the SF and the control groups at the phylum, genus and species levels. Notably, the *Proteobacteria* phylum, *Neisseria* genus, and *Neisseria sicca* species were more abundant in the SF group. In contrast, microbes characterized by ultra-small cell size, reduced genomes, limited biosynthetic capabilities and a parasitic lifestyle [17], such as those from the *Candidatus saccharibacteria* phylum, *Candidatus Saccharibacteri unclassified* genus and *Candidatus Nanosynsaccharibacteria sp. TM7 ANC 38.39 G1 1* species were more abundant in the control group compared to the SF group (Figure 4A–C, Supplementary Figures S2–S4). Taken together, the heatmap suggests that SF is associated with a distinct plaque biofilm microbial composition.

Out of 354 species, 324 species were common to both the SF and control groups. Seventeen species were exclusively found in the SF group, and these belonged to the five prokaryotic phyla viz, *Actinobacteria* (6 species), followed by *Bacteroidetes* (5 species), *Firmicutes* (2 species), *Proteobacteria* (2 species), *Fusobacteria* (1 species), and a single archea group organism belonging to *Euryarchaeota* (1 species) (Supplementary Table S2). In contrast, 13 species were unique to the control group and comprised the phyla *Firmicutes* (6 species), *Bacteroidetes* (3 species), *Candidatus Saccharibacteria* (2 species), *Proteobacteria* (1 species), and *Synergistetes* (1 species) (Figure 4D, Supplementary Table S3).

**Figure 5.**
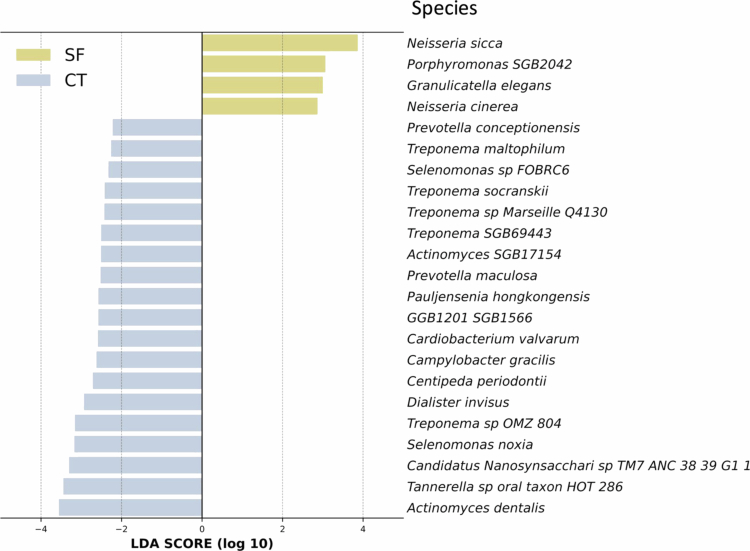
Linear discriminant analysis effect size (LEfSe) of microbial composition in the severe dental fluorosis (SF) and control (CT) groups. LEfSe analysis at the species level identified microbial biomarkers with an LDA score (log₁₀) > 2 and *p*-value < 0.05. Four species were significantly enriched in the SF group: *Neisseria sicca*, *Granulicatella elegans*, *Porphyromonas* SGB2042 and *Neisseria cinerea*. In the control group, the five species with the highest LDA scores were *Actinomyces dentalis*, *Tanner Ella* sp. oral taxon HOT 286, *Candidatus Nanosynsacchari* sp. TM7 ANC 38.39 G1 1, *Selenomonas noxia* and *Treponema sp OMZ 804.* CT = control, SF = severe fluorosis.

We conducted LEfSe analysis to identify significant differences in species between the SF and control groups, using an LDA score (log10) > 2 and a *p*-value < 0.05 as criteria. Four species were significantly enriched in the SF group, and these were *Neisseria sicca*, *Granulicatella elegans, Porphyromonas* SGB2042*, and Neisseria cinerea*. In the control group, nineteen species were significantly more abundant, and these included *Actinomyces dentalis, Tannerella sp. oral taxon HOT 286*, *Candidatus Nanosynsacchari sp. TM7 ANC 38.39 G1 1*, *Selenomonas noxia*, and *Treponema sp OMZ 804,* which were the top five most predominant species with the highest LDA scores (Figure 5, Supplementary Table S4).

### Metabolic profiles

**Figure 6.**
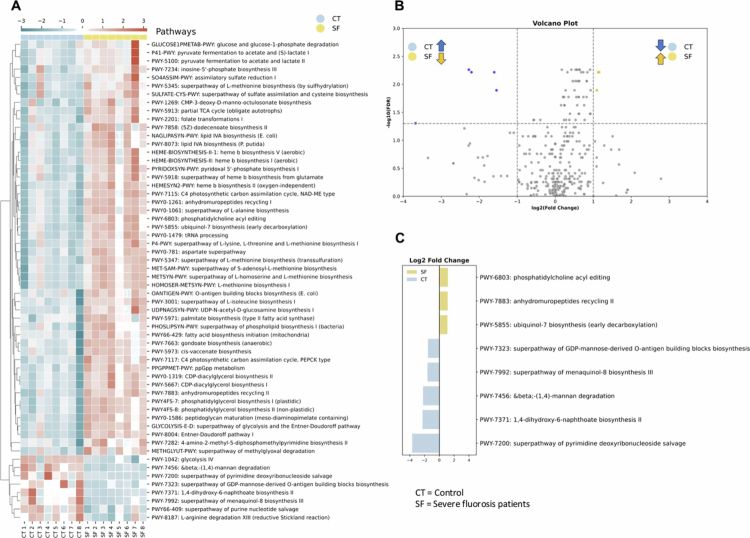
Heatmap analysis and volcano plot of metabolic pathway profiles. (A) Heatmap showing 58 significantly different pathways (FDR < 0.05) between the severe fluorosis (SF) and control (CT) groups. (B) Volcano plot highlighting dominant pathways in the SF and CT groups, defined by FDR < 0.05 and a fold change greater than two (|log2FC| > 1). (C) Log2 fold change of the eight most discriminative pathways, illustrating differential expression between groups. CT = control, SF = severe fluorosis.

The relative abundance of functional metabolic pathways in the SF and control groups was analyzed to compare their composition. Out of 353 mapped metabolic pathways, 58 showed significantly different relative abundance between the SF and control groups (Benjamini–Hochberg FDR < 0.05) (Supplementary Table S5). A heatmap of these pathways demonstrated distinct expression patterns across groups (Figure 6A). A volcano plot highlighted seven significantly differentially expressed pathways with a fold change greater than 2 (log2FC > 1 or log2FC < –1) among the 58 significant pathways (Figure 6B). Of these, three pathways were overexpressed in the SF group comprising phosphatidylcholine acyl editing (log2FC = 1.16, FDR < 0.01), anhydromuropeptides recycling II (log2FC = 1.13, FDR < 0.01), and ubiquinol-7 biosynthesis (log2FC = 1.09, FDR < 0.05), all of which were attributed to *N. sicca* (Supplementary Table S6).

Five pathways were overexpressed in the control group: the superpathway of pyrimidine deoxyribonucleoside salvage (log2FC = –3.68, FDR < 0.05), (1,4)-dihydroxy-6-naphthoate biosynthesis II (log2FC = –2.27, FDR < 0.01), *β*-(1,4)-mannan degradation (log2FC = –2.21, FDR < 0.05), the superpathway of menaquinol-8 biosynthesis II (log2FC = –1.61, FDR < 0.01), and the superpathway of GDP-mannose-derived O-antigen building blocks biosynthesis (log2FC = –1.55, FDR < 0.05) (Figure 6C, Supplementary Table S5).

## Discussion

While the detrimental effects of DF on the appearance and the physical quality of enamel are well-documented, its impact on the oral microbiome remains unclear. To further study this phenomenon, we employed shotgun metagenomic, next generation sequencing (Illumina Novaseq), a comprehensive and less biased approach than 16S rRNA sequencing [18,19], to analyze the supragingival biofilm of SF with controls. This permitted a detailed analysis of microbial composition and functional metabolomics of the biofilm in a fluorotic milieu.

Beta diversity metrics of our study revealed distinct microbial communities between groups, suggesting that DF may impact species diversity and their relative abundance. Of the 354 identified species, 324 were present in both the SF and control groups and both groups shared the identical five predominant core phyla, consistent with prior studies of the healthy oral microbiome [20–22]. However, the proportions of the core phyla differed between the two groups.

In a previous study [23] reported salivary microbiome shifts in moderate/severe fluorosis and noted increased Firmicutes, and decreased Bacteroidetes genera [23]. In contrast, our analysis of plaque biofilm from SF revealed decreased levels of *Firmicutes*, *Bacteroidetes* and *Actinobacteria*, alongside increased levels *Proteobacteria*, relative to controls. These disparities likely reflect differences in sample source (saliva, biofilm), the degree of fluorosis, or the analytical techniques.

The observed shift in phylum-level predominance from *Actinobacteria* and *Firmicutes* in the control group to *Proteobacteria* predominance in the SF group indicates a significant fluorosis-induced alteration of the plaque biofilm. This shift is further evidenced by the transition in dominant genera from *Actinomyces* in controls to *Neisseria* in the SF group. The extent of this microbial restructuring is highlighted by the absence of shared species among the top five most abundant species, with *Actinomyces dentalis* leading in controls and *Neisseria sicca* dominating in SF.

*N. sicca* was the most abundant species in all cases of SF. Although typically a commensal organism abundant in healthy plaque [24,25] its increased abundance in SF suggests a specific adaptation to the high-fluoride environment*. N. sicca,* a putative probiotic [26], also exhibits high alcohol dehydrogenase activity, converting ethanol to carcinogenic acetaldehydes [27,28], although this might also neutralize an acidic milieu. We hypothesize that the dominance of *N. sicca* in SF is driven by a combination of biochemical resistance and physical niche adaptation. Firstly, *N. sicca* may possess a higher intrinsic resistance to the antimicrobial effects of fluoride. This is supported by literature showing that it possesses a fluoride-inhibitory serine esterase [29], providing a direct mechanism to thrive in a high-fluoride environment that might inhibit other competing species. Secondly, the altered niche formation due to the physical modifications of the enamel surface in SF (e.g. modifications to the enamel surface in SF, such as increased porosity and pitting) could result in an altered niche formation. This new microenvironment may preferentially favor the adhesion and proliferation of *N. sicca* over other commensals adapted to smooth enamel. Together, these factors could explain the observed ecological shift. Additionally, species significantly enriched in SF were *Neisseria cinerea* (an opportunistic pathogen [30]), acidogenic *Granulicatella elegans* [31], and *Porphyromonas* SGB2042 [32]. These findings reveal a shift toward *Proteobacterial* dominance in fluorosis, highlighting complex microbial dynamics that warrant further investigation of their specific roles and fluoride adaptation mechanisms.

On the other hand, species such as *Actinomyces dentalis*, *Candidatus Nanosynbacter* sp. TM7 ANC 38.39 G1 1, *Tannerella* sp. oral taxon HOT 286, *Selenomonas noxia* and *Treponema sp. OMZ 804* were significantly enriched in the control group. *A. dentalis* is a well-known early colonizer that initiates dental biofilm formation [33]. *Candidatus Nanosynbacter* sp. TM7 is an ultra-small, parasitic oral organism from the phylum *Candidatus Saccharibacteria* with a reduced genome. It exists in an obligate episymbiotic relationship with its host, *Schaalia odontolytica* (formerly *Actinomyces odontolyticus* strain XH001) [34]. These organisms are abundant in healthy supragingival biofilms [17,35].

Unlike the keystone, periodontopathogen *Tannerella forsythia* [35,36], *Tannerella sp. oral taxon HOT 286* is considered a health-associated species. This distinction is supported by the genetic differences between the virulent and the commensal counterparts such as the gene/GC content, synteny, and metabolic pathways. Notably, *HOT 286* lacks or has altered versions of key *T. forsythia* virulence genes like *karilysin*, *prtH* and *bspA*, which mediate adhesion, colonization, and virulence [36]. The absence of these factors likely contributes to the benign nature of *HOT 286* in the oral microbiome. Furthermore, our findings also support beneficial roles for *Selenomonas noxia.* The abundance of this species in healthy supragingival biofilm is congruent with its role in eubiotic oral eco-biome [37,38].

In terms of the pathway analysis significant alterations in biosynthesis and degradation pathways were notable in SF biofilms relative to the controls. Upregulation of phosphatidylcholine (PC) acyl editing in SF, a key membrane lipid biosynthesis cycle [39], likely reflects a response to fluoride-induced membrane disruption, as fluorosis is known to decrease PC content and impair cellular function [40,41]. Conversely, GDP-sugar biosynthesis, crucial for O-antigen synthesis in Gram-negative outer membranes [42], was downregulated in SF, suggesting fluoride interference with this membrane-related pathway. Furthermore, three upregulated quinol/quinone biosynthesis pathways were prominent: ubiquinol-7 biosynthesis, menaquinol-8 biosynthesis III (superpathway), and 1,4-dihydroxy-6-naphthoate biosynthesis II. Ubiquinol-7 (CoQ), synthesized by *Neisseria*, is vital for electron transport and ATP production, impacting cellular homeostasis [43–45]. Menaquinol-8 (vitamin K2), derived via 1,4-dihydroxy-6-naphthoate, aids aerobic respiration and stress survival [46,47]. The upregulation of these pathways, particularly ubiquinol-7, likely supports the survival and growth of the enriched *Neisseria* species (*N. sicca*, *N. cinerea*) within the challenging SF eco-biome. Among degradation pathways, anhydromuropeptides recycling II (peptidoglycan recycling crucial for cell wall integrity, stress response, and antibiotic resistance [48,49] was significantly upregulated in the SF groups. Notably, *N. sicca* was the primary species associated with this pathway, suggesting its upregulation enhancing its survival and resistance in the challenging SF environment.

Finally, our previous work on salivary proteomic signatures in SF adds to the current data by identifying altered salivary proteins associated with the condition. In particular, we noted increased CFTR-linked proteins, such as LDHA, UBA1, implying that the latter may play a role in fluorosis [2]. Together current findings on the microbiome and the metabolome of the SF plaque enhance our understanding on the effect of excess fluoride on the oral eco-biome providing valuable insights for future researchers.

Our study has several limitations. These include potential confounders from multiple sources of fluoride exposure, the unknown duration of high fluoride exposure in the cohorts, and severity of their enamel defects all of which could impact the microbiome. Additionally, unknown host immune or epigenetic responses to fluoride may contribute to the observed microbial shift. The study's generalizability is limited by its focus on two endemic geographic locations, as fluorosis severity and microbial composition can vary regionally. Factors intrinsic to these areas, such as local dietary patterns, lifestyle, geography and socioeconomic status, may serve as unmeasured confounders that influenced the observed microbial profiles. Furthermore, the small sample size may have limited our power to detect more subtle microbial differences. Finally, while participants were matched by age, they were not matched by sex due to limited sample availability. We prioritized age matching, a critical variable in both developmental and microbiome studies, consistent with prior work [23]. Although recent evidence suggests sex has a minimal influence on fluorosis risk [50], we acknowledge that potential sex-based biological variation remains a limitation of this study.

Another limitation of this study is its cross-sectional design, which captures a single time point and thus restricts the ability to infer causality. To overcome this, an ideal future direction would be a prospective birth cohort study, initiated prior to significant fluoride exposure. This longitudinal design would enable the concurrent monitoring of microbial succession, cumulative fluoride exposure, and enamel development across key mineralization stages. Such a study would be instrumental in moving beyond the associations observed here to elucidate potential causal relationships and to identify early microbial biomarkers predictive of fluorosis risk. While this longitudinal approach represents the gold standard, it was not feasible for the present study due to significant practical constraints, such as long-term participant retention and precise exposure quantification. Our cross-sectional analysis provides a foundational snapshot of the microbial communities associated with SF.

## Conclusions

This study demonstrates a significant shift in the microbiota associated with SF. The most striking feature of the study was the emergence of *N. sicca* as the dominant microbial species in SF, suggesting it may be a key driver of the microbial imbalance. This was accompanied by a significant phylum-level shift from *Actinobacteria* (controls) to *Proteobacteria* (SF). Specifically, SF was characterized by increased prevalence of potentially detrimental taxa: *Neisseria cinerea* (opportunistic pathogen), *Granulicatella elegans* (acidogenic, immuno-modulatory), and *Porphyromonas* SGB2042 (periodontally associated). Conversely, species that were abundant in controls but were depleted in SF, such as *Actinomyces dentalis* and *Candidatus Nanosynbacter* sp. TM7, were indicative of a healthy biofilm formation and microbial interactions. Functionally, key metabolic pathways upregulated in SF – phosphatidylcholine acyl editing, anhydromuropeptides recycling II, and ubiquinol-7 biosynthesis – likely facilitate *N. sicca*'s survival and adaptation in a fluoride-rich environment.

We interpret the enrichment of *N. sicca* in SF as a situational biomarker, reflecting a microbial adaptation to the altered oral niche (e.g. high fluoride levels and enamel porosity), rather than a direct etiological driver of fluorosis development during tooth formation. Consequently, its utility as an early diagnostic or predictive biomarker for fluorosis requires further validation through future longitudinal studies.

From a therapeutic perspective, our functional pathway analysis suggests promising avenues for microbiome-targeted interventions. Strategies involving prebiotics, probiotics, or novel fluoride formulations could be designed to restore microbial balance and limit the overgrowth of adaptive species like *N. sicca.* While these interventions would not prevent the initial development of fluorosis, they hold significant potential for high-risk populations. Such approaches could mitigate the progression of secondary issues related to dental fluorosis and reduce the co-occurrence or exacerbation of other oral conditions, including dental caries, periodontal disease, and oral candidiasis, which may arise from compromised enamel integrity and dysbiotic shifts in the oral microbiome. By addressing these broader microbial implications, such interventions offer a pathway toward more holistic oral health management for individuals affected by fluorosis.

## Supplementary Material

Supplementary material **Supplementary Table S1**. The average relative abundance in different taxonomic levels found in the severe fluorosis group (SF) and control group.**Supplementary Table S2**. Microbes exclusively found in the severe fluorosis group.**Supplementary Table S3**. Microbes exclusively found in the control group.**Supplementary Table S4**.Microbes with significantly different abundances between the severe fluorosis (SF) and the control (CT) groups.**Supplementary Table S5**. Statistically significant pathways between the severe fluorosis (SF) and the control (CT) groups.**Supplementary Table S6**. Species-stratified functional pathway abundances from the HUMAnN3 pipeline. The table shows the abundance of each metabolic pathway attributed to individual contributing species.**Supplementary Figure S1**. Rarefaction analysis of expected gene family and sequencing depth. The plot shows the number of unique gene families observed (expected gene family) as a function of increasing sequencing depth (million reads). Each line represents an individual sample from either the control (CT) or severe fluorosis (SF) group.**Supplementary Figure S2**. Heatmap of the 20 most abundant phyla across individual subjects. The relative abundance data for each phylum was *z*-score normalized. The color key represents the *z*-score values. CT = control, SF = severe fluorosis.**Supplementary Figure S3**. Heatmap of the 20 most abundant genera across individual subjects. The relative abundance data for each genus was *z*-score normalized. The color key represents the *z*-score values. CT = control, SF = severe fluorosis.

## Data Availability

All oral microbiome data from sequencing were deposited on the National Center for Biotechnology Information by Sequence Read Archive (SRA) in BioProject number PRJNA1217055 or linked to https://www.ncbi.nlm.nih.gov/sra/PRJNA1217055.
