# Supplementary material for: The metagenome and metabolome signatures of dental biofilms associated with severe dental fluorosis
Source: J Oral Microbiol. 2025 Sep 23;17(1):2560591. doi: 10.1080/20002297.2025.2560591 (PMC12459186; doi:10.1080/20002297.2025.2560591)
Supplement: Supplementary material — Supplementary Table S1. The average relative abundance in different taxonomic levels found in the severe fluorosis group (SF) and control group.Supplementary Table S2. Microbes exclusively found in the severe fluorosis group.Supplementary Table S3. Microbes exclusively found in the control group.Supplementary Table S4.Microbes with significantly different abundances between the severe fluorosis (SF) and the control (CT) groups.Supplementary Table S5. Statistically significant pathways between the severe fluorosis (SF) and the control (CT) groups.Supplementary Table S6. Species-stratified functional pathway abundances from the HUMAnN3 pipeline. The table shows the abundance of each metabolic pathway attributed to individual contributing species.Supplementary Figure S1. Rarefaction analysis of expected gene family and sequencing depth. The plot shows the number of unique gene families observed (expected gene family) as a function of increasing sequencing depth (million reads). Each line represents an individual sample from either the control (CT) or severe fluorosis (SF) group.Supplementary Figure S2. Heatmap of the 20 most abundant phyla across individual subjects. The relative abundance data for each phylum was z-score normalized. The color key represents the z-score values. CT = control, SF = severe fluorosis.Supplementary Figure S3. Heatmap of the 20 most abundant genera across individual subjects. The relative abundance data for each genus was z-score normalized. The color key represents the z-score values. CT = control, SF = severe fluorosis. [file ZJOM_A_2560591_SM5831.docx]

**Supplementary Data**

**Supplementary Table S1. The average relative abundance in different taxonomic levels found in the severe fluorosis group (SF) and control group.**

| **No.** | **Taxonomic levels** | **Group** | **Relative abundance** |
| --- | --- | --- | --- |
| 1 | P_Proteobacteria | SF | 39.70 ± 7.70 |
| 2 | P_Firmicutes | SF | 20.32 ± 9.48 |
| 3 | P_Actinobacteria | SF | 15.83 ± 11.66 |
| 4 | *G_Neisseria* | SF | 22.00 ± 4.76 |
| 5 | *G_Streptococcus* | SF | 10.53 ± 6.28 |
| 6 | *G_Actinomyces* | SF | 7.11 ± 4.83 |
| 7 | *S_Neisseria sicca* | SF | 13.47 ± 5.38 |
| 8 | *S_ Lautropia mirabilli* | SF | 5.80 ± 3.87 |
| 9 | *S_ Streptococcus sanguinis* | SF | 4.92 ± 2.27 |
| 10 | *S_ Haemophilus parainfluenzae* | SF | 4.54 ± 3.06 |
| 11 | *S_ Neisseria elongata* | SF | 4.13 ± 3.15 |
| 12 | P_Actinobacteria | Control | 25.19 ± 11.78 |
| 13 | P_Firmicutes | Control | 21.78 ± 7.99 |
| 14 | P_Proteobacteria | Control | 16.82 ± 6.57 |
| 15 | *G_Actinomyces* | Control | 16.51 ± 7.27 |
| 16 | *G_Neisseria* | Control | 7.15 ± 43.91 |
| 17 | *G_Tannerella* | Control | 5.45 ± 3.77 |
| 18 | *S_ Actinomyces dentalis* | Control | 6.60 ± 3.65 |
| 19 | *S_ Tannerella sp. oral taxon HOT 286* | Control | 5.23 ± 3.70 |
| 20 | *S_ Candidatus Nanosynsacchari sp. TM7 ANC 38.39 G1 1* | Control | 3.64 ± 2.04 |
| 21 | *S_ Porphyromonas pasteri* | Control | 3.37 ± 2.96 |
| 22 | *S_ Actinomyces naeslundii* | Control | 3.25 ± 2.49 |

**Supplementary Table S2. Microbes exclusively found in the severe fluorosis group**.

| **No.** | **Species** | **Phylum** |
| --- | --- | --- |
| 1 | *Actinomyces_sp_S6_Spd3* | Actinobacteria |
| 2 | *Olsenella_phocaeensis* | Actinobacteria |
| 3 | *Atopobium_sp_oral_taxon_199* | Actinobacteria |
| 4 | *Propionibacterium_acidifaciens* | Actinobacteria |
| 5 | *GGB39918_SGB47522* | Actinobacteria |
| 6 | *Actinomyces_SGB15893* | Actinobacteria |
| 7 | *Methanobrevibacter_oralis* | Archaea |
| 8 | *Prevotella_histicola* | Bacteriodetes |
| 9 | *Alloprevotella_SGB1466* | Bacteriodetes |
| 10 | *GGB1022_SGB1316* | Bacteriodetes |
| 11 | *Prevotella_sp_oral_taxon_376* | Bacteriodetes |
| 12 | *GGB1186_SGB1534* | Bacteriodetes |
| 13 | *GGB3886_SGB5269* | Firmicutes |
| 14 | *GGB51111_SGB71327* | Firmicutes |
| 15 | *Fusobacterium_massiliense* | Fusobacteria |
| 16 | *GGB2670_SGB3597* | Proteobacteria |
| 17 | *Simonsiella_muelleri* | Proteobacteria |

**Supplementary Table S3. Microbes exclusively found in the control group.**

| **No.** | **Species** | **Phylum** |
| --- | --- | --- |
| 1 | *Prevotella_multiformis* | Bacteriodetes |
| 2 | *Prevotella_salivae* | Bacteriodetes |
| 3 | *GGB1202_SGB1567* | Bacteriodetes |
| 4 | *GGB12763_SGB19797* | Candidatus_Saccharibacteria |
| 5 | *Candidatus_Saccharibacteria_unclassified_SGB19782* | Candidatus_Saccharibacteria |
| 6 | *Streptococcus_sobrinus* | Firmicutes |
| 7 | *Oribacterium_parvum* | Firmicutes |
| 8 | *GGB4308_SGB5895* | Firmicutes |
| 9 | *Bacillus_velezensis* | Firmicutes |
| 10 | *GGB4721_SGB6537* | Firmicutes |
| 11 | *Peptostreptococcaceae_bacterium_oral_taxon_113* | Firmicutes |
| 12 | *Desulfovibrio_sp_Dsv1* | Proteobacteria |
| 13 | *Pyramidobacter_piscolens* | Synergistetes |

**Supplementary Table S4. Microbes with significantly different abundances between the severe fluorosis (SF) and the control (CT) groups.**

| **No.** | **Species** | **Group**  **enriched** | **Average RA: CT** | **Average RA: SF** | **SD**  **of CT** | **SD**  **of SF** | **LDA**  **score** | ***p-value*** |
| --- | --- | --- | --- | --- | --- | --- | --- | --- |
| 1 | *Neisseria sicca* | Patient | 2.5861 | 13.4732 | 1.7944 | 5.3831 | 3.8701 | 0.0011 |
| 2 | *Porphyromonas SGB2042* | Patient | 0.0006 | 0.0073 | 0.0017 | 0.0111 | 3.0678 | 0.0206 |
| 3 | *Granulicatella elegans* | Patient | 0.0017 | 0.0107 | 0.0018 | 0.0083 | 3.0028 | 0.0173 |
| 4 | *Neisseria cinerea* | Patient | 0.0004 | 0.0236 | 0.0010 | 0.0233 | 2.8740 | 0.0020 |
| 5 | *Prevotella conceptionensis* | Control | 0.2079 | 0.0384 | 0.1438 | 0.0256 | 2.2246 | 0.0046 |
| 6 | *Treponema maltophilum* | Control | 0.2663 | 0.0337 | 0.2483 | 0.0178 | 2.2644 | 0.0033 |
| 7 | *Selenomonas_sp FOBRC6* | Control | 0.2569 | 0.0499 | 0.1734 | 0.0934 | 2.3291 | 0.0087 |
| 8 | *Treponema_socranskii* | Control | 0.4626 | 0.0376 | 0.4488 | 0.0262 | 2.4223 | 0.0016 |
| 9 | *Treponema_sp_Marseille_Q4130* | Control | 0.0260 | 0.0010 | 0.0356 | 0.0009 | 2.4321 | 0.0023 |
| 10 | *Treponema_SGB69443* | Control | 0.0248 | 0.0030 | 0.0196 | 0.0026 | 2.5105 | 0.0063 |
| 11 | *Actinomyces_SGB17154* | Control | 0.0450 | 0.0028 | 0.0425 | 0.0044 | 2.5151 | 0.0405 |
| 12 | *Prevotella_maculosa* | Control | 0.2509 | 0.0399 | 0.1284 | 0.0335 | 2.5261 | 0.0087 |
| 13 | *Pauljensenia_hongkongensis* | Control | 0.5759 | 0.1526 | 0.2477 | 0.0861 | 2.5780 | 0.0023 |
| 14 | *GGB1201_SGB1566* | Control | 0.0206 | 0.0003 | 0.0234 | 0.0006 | 2.5799 | 0.0014 |
| 15 | *Cardiobacterium_valvarum* | Control | 0.8771 | 0.2532 | 0.7598 | 0.1669 | 2.5926 | 0.0357 |
| 16 | *Campylobacter_gracilis* | Control | 0.6294 | 0.1694 | 0.4257 | 0.1120 | 2.6285 | 0.0063 |
| 17 | *Centipeda_periodontii* | Control | 0.0403 | 0.0080 | 0.0239 | 0.0096 | 2.7171 | 0.0045 |
| 18 | *Dialister invisus* | Control | 1.1942 | 0.0784 | 2.1998 | 0.0657 | 2.9370 | 0.0023 |
| 19 | *Treponema_sp_OMZ_804* | Control | 0.0086 | 0.0020 | 0.0059 | 0.0028 | 3.1632 | 0.0229 |
| 20 | *Selenomonas_noxia* | Control | 2.5101 | 0.2424 | 1.9050 | 0.1329 | 3.1745 | 0.0008 |
| 21 | *Candidatus_Nanosynsacchari_sp_TM7_ANC_38_39_G1_1* | Control | 3.6356 | 1.0827 | 2.0353 | 0.8680 | 3.3077 | 0.0117 |
| 22 | *Tannerella_sp_oral_taxon_HOT_286* | Control | 5.2329 | 0.8608 | 3.6987 | 0.4154 | 3.4481 | 0.0008 |
| 23 | *Actinomyces_dentalis* | Control | 6.5960 | 1.5074 | 3.6453 | 0.9731 | 3.5608 | 0.0016 |

Abbreviations: CT = Control, SF =Severe Fluorosis, RA = Relative abundance

**Supplementary Table S5. Statistically significant pathways between the severe fluorosis (SF) and the control (CT) groups.**

| **Pathway** | **Mean CT** | **Mean SF** | **Std CT** | **Std SF** | **log2fc** | **FDR** |
| --- | --- | --- | --- | --- | --- | --- |
| **PWY-7371: 1,4-dihydroxy-6-naphthoate biosynthesis II** | 67.5838 | 13.9706 | 40.1362 | 4.8652 | -2.2743 | 0.0055 |
| **PWY-3001: superpathway of L-isoleucine biosynthesis I** | 426.1688 | 480.4891 | 32.1207 | 14.7909 | 0.1731 | 0.0055 |
| **PWY0-781: aspartate superpathway** | 221.7021 | 297.5511 | 27.7432 | 18.1214 | 0.4245 | 0.0055 |
| **PWY-7117: C4 photosynthetic carbon assimilation cycle, PEPCK type** | 216.0638 | 312.4210 | 37.8838 | 35.2203 | 0.5320 | 0.0055 |
| **PWY0-1319: CDP-diacylglycerol biosynthesis II** | 421.2523 | 623.0826 | 86.2624 | 66.8358 | 0.5647 | 0.0055 |
| **PWY-5667: CDP-diacylglycerol biosynthesis I** | 413.4548 | 614.1395 | 83.1497 | 68.6393 | 0.5708 | 0.0055 |
| **HEMESYN2-PWY: heme b biosynthesis II (oxygen-independent)** | 227.4051 | 349.2020 | 37.1235 | 31.3382 | 0.6188 | 0.0055 |
| **PWY4FS-7: phosphatidylglycerol biosynthesis I (plastidic)** | 297.6733 | 462.9409 | 64.8846 | 32.7022 | 0.6371 | 0.0055 |
| **PWY4FS-8: phosphatidylglycerol biosynthesis II (non-plastidic)** | 296.3659 | 461.1026 | 64.3957 | 32.2052 | 0.6377 | 0.0055 |
| **PPGPPMET-PWY: ppGpp metabolism** | 109.8419 | 207.3054 | 36.8408 | 27.2562 | 0.9163 | 0.0055 |
| **PWY-7456: &beta;-(1,4)-mannan degradation** | 17.7627 | 3.8523 | 6.6475 | 1.6124 | -2.2051 | 0.0061 |
| **PWY-7992: superpathway of menaquinol-8 biosynthesis III** | 61.5499 | 20.2232 | 28.5930 | 9.7145 | -1.6057 | 0.0061 |
| **UDPNAGSYN-PWY: UDP-N-acetyl-D-glucosamine biosynthesis I** | 511.5489 | 638.6148 | 50.2502 | 64.5620 | 0.3201 | 0.0061 |
| **GLYCOLYSIS-E-D: superpathway of glycolysis and the Entner-Doudoroff pathway** | 187.0880 | 300.2886 | 50.7619 | 32.0180 | 0.6826 | 0.0061 |
| **PWY0-1586: peptidoglycan maturation (meso-diaminopimelate containing)** | 609.0346 | 1015.2765 | 153.9782 | 101.5557 | 0.7373 | 0.0061 |
| **PWY0-1261: anhydromuropeptides recycling I** | 183.5388 | 329.5651 | 54.3557 | 39.2472 | 0.8445 | 0.0061 |
| **PWY-7883: anhydromuropeptides recycling II** | 125.3552 | 274.8589 | 58.4162 | 32.5769 | 1.1327 | 0.0061 |
| **PWY-6803: phosphatidylcholine acyl editing** | 82.0505 | 182.8471 | 36.4696 | 32.1041 | 1.1561 | 0.0061 |
| **MET-SAM-PWY: superpathway of S-adenosyl-L-methionine biosynthesis** | 231.8310 | 318.8999 | 42.5946 | 26.6467 | 0.4600 | 0.0088 |
| **METSYN-PWY: superpathway of L-homoserine and L-methionine biosynthesis** | 225.7610 | 325.1483 | 46.6319 | 25.5219 | 0.5263 | 0.0088 |
| **PWY-5347: superpathway of L-methionine biosynthesis (transsulfuration)** | 209.8380 | 312.5773 | 43.9077 | 18.5936 | 0.5749 | 0.0088 |
| **PWY-7115: C4 photosynthetic carbon assimilation cycle, NAD-ME type** | 156.1863 | 239.6861 | 31.5404 | 28.2854 | 0.6179 | 0.0088 |
| **HOMOSER-METSYN-PWY: L-methionine biosynthesis I** | 156.1433 | 242.8079 | 38.4093 | 21.3369 | 0.6369 | 0.0088 |
| **PWY-5913: partial TCA cycle (obligate autotrophs)** | 147.9249 | 262.2191 | 40.0103 | 43.2496 | 0.8259 | 0.0088 |
| **PWY0-1061: superpathway of L-alanine biosynthesis** | 183.9010 | 326.8268 | 49.7553 | 44.9390 | 0.8296 | 0.0088 |
| **PWY-7323: superpathway of GDP-mannose-derived O-antigen building blocks biosynthesis** | 46.8628 | 16.0530 | 22.6667 | 6.8622 | -1.5456 | 0.0128 |
| **PWY-7663: gondoate biosynthesis (anaerobic)** | 490.9021 | 592.6811 | 60.1544 | 33.2842 | 0.2718 | 0.0128 |
| **P4-PWY: superpathway of L-lysine, L-threonine and L-methionine biosynthesis I** | 230.6320 | 327.7396 | 44.2231 | 33.3441 | 0.5070 | 0.0128 |
| **HEME-BIOSYNTHESIS-II-1: heme b biosynthesis V (aerobic)** | 146.6277 | 279.6388 | 47.0239 | 62.3501 | 0.9314 | 0.0128 |
| **PWY-5855: ubiquinol-7 biosynthesis (early decarboxylation)** | 83.8637 | 179.0771 | 38.1407 | 33.7464 | 1.0945 | 0.0128 |
| **PWY-5973: cis-vaccenate biosynthesis** | 507.1581 | 584.5123 | 56.6592 | 25.8997 | 0.2048 | 0.0169 |
| **P41-PWY: pyruvate fermentation to acetate and (S)-lactate I** | 445.8679 | 522.4875 | 31.8947 | 66.2007 | 0.2288 | 0.0169 |
| **PWY-5100: pyruvate fermentation to acetate and lactate II** | 445.8679 | 522.4875 | 31.8947 | 66.2007 | 0.2288 | 0.0169 |
| **PHOSLIPSYN-PWY: superpathway of phospholipid biosynthesis I (bacteria)** | 317.4088 | 404.0239 | 52.3573 | 32.9643 | 0.3481 | 0.0169 |
| **PYRIDOXSYN-PWY: pyridoxal 5'-phosphate biosynthesis I** | 250.5548 | 368.1460 | 59.9777 | 57.5341 | 0.5552 | 0.0169 |
| **NAGLIPASYN-PWY: lipid IVA biosynthesis (E. coli)** | 208.0094 | 318.9131 | 50.4386 | 50.9028 | 0.6165 | 0.0169 |
| **PWY-8073: lipid IVA biosynthesis (P. putida)** | 208.0094 | 318.9131 | 50.4386 | 50.9028 | 0.6165 | 0.0169 |
| **PWY0-1479: tRNA processing** | 288.8493 | 482.4863 | 88.9254 | 73.2941 | 0.7402 | 0.0169 |
| **HEME-BIOSYNTHESIS-II: heme b biosynthesis I (aerobic)** | 157.8634 | 279.6388 | 43.1864 | 62.3501 | 0.8249 | 0.0169 |
| **OANTIGEN-PWY: O-antigen building blocks biosynthesis (E. coli)** | 488.4276 | 543.1301 | 40.4837 | 21.0861 | 0.1532 | 0.0237 |
| **PWY66-429: fatty acid biosynthesis initiation (mitochondria)** | 448.7784 | 530.9833 | 63.0981 | 33.3649 | 0.2427 | 0.0237 |
| **PWY-7282: 4-amino-2-methyl-5-diphosphomethylpyrimidine biosynthesis II** | 253.8635 | 300.7823 | 32.1145 | 27.4376 | 0.2447 | 0.0237 |
| **PWY-5918: superpathway of heme b biosynthesis from glutamate** | 232.4031 | 346.4593 | 62.6107 | 52.1863 | 0.5761 | 0.0237 |
| **PWY-8004: Entner-Doudoroff pathway I** | 165.5748 | 280.5641 | 52.8945 | 23.2330 | 0.7608 | 0.0237 |
| **PWY-5345: superpathway of L-methionine biosynthesis (by sulfhydrylation)** | 192.5193 | 251.8786 | 43.7254 | 31.2852 | 0.3877 | 0.0336 |
| **PWY-5971: palmitate biosynthesis (type II fatty acid synthase)** | 298.3537 | 456.3159 | 121.7529 | 84.5667 | 0.6130 | 0.0336 |
| **PWY-7858: (5Z)-dodecenoate biosynthesis II** | 163.8726 | 253.1709 | 40.1214 | 60.1411 | 0.6275 | 0.0336 |
| **PWY-2201: folate transformations I** | 152.6212 | 287.6346 | 76.3750 | 58.5725 | 0.9143 | 0.0336 |
| **SO4ASSIM-PWY: assimilatory sulfate reduction I** | 122.1055 | 231.9193 | 58.9094 | 92.1379 | 0.9255 | 0.0336 |
| **PWY66-409: superpathway of purine nucleotide salvage** | 316.9690 | 213.2930 | 58.9404 | 62.8108 | -0.5715 | 0.0433 |
| **PWY-8187: L-arginine degradation XIII (reductive Stickland reaction)** | 392.3805 | 266.6689 | 79.3223 | 76.1407 | -0.5572 | 0.0433 |
| **PWY-1042: glycolysis IV** | 590.8601 | 450.0171 | 68.5364 | 49.5843 | -0.3928 | 0.0433 |
| **PWY-7234: inosine-5'-phosphate biosynthesis III** | 452.4175 | 530.9995 | 64.8542 | 44.7909 | 0.2311 | 0.0433 |
| **GLUCOSE1PMETAB-PWY: glucose and glucose-1-phosphate degradation** | 266.5826 | 323.9781 | 27.4607 | 59.2015 | 0.2813 | 0.0433 |
| **PWY-1269: CMP-3-deoxy-D-manno-octulosonate biosynthesis** | 191.0523 | 247.0006 | 32.1523 | 25.4805 | 0.3705 | 0.0433 |
| **SULFATE-CYS-PWY: superpathway of sulfate assimilation and cysteine biosynthesis** | 179.9068 | 260.5705 | 55.1036 | 37.2998 | 0.5344 | 0.0433 |
| **METHGLYUT-PWY: superpathway of methylglyoxal degradation** | 80.1019 | 136.9608 | 29.3942 | 29.9568 | 0.7739 | 0.0433 |
| **PWY-7200: superpathway of pyrimidine deoxyribonucleoside salvage** | 29.5861 | 2.3138 | 22.0590 | 2.4289 | -3.6766 | 0.0497 |

**Supplementary Table S6. Species-stratified functional pathway abundances from the HUMAnN3 pipeline.** The table shows the abundance of each metabolic pathway attributed to individual contributing species.

| **Pathways** | **Species** |
| --- | --- |
| PWY-5855: ubiquinol-7 biosynthesis (early decarboxylation) | *Aggregatibacter aphrophilus* |
|  | *Eikenella corrodens* |
|  | *Eikenella sp NML130454* |
|  | *Kingella denitrificans* |
|  | *Kingella oralis* |
|  | *Morococcus cerebrosus* |
|  | *Neisseria bacilliformis* |
|  | *Neisseria cinerea* |
|  | *Neisseria elongata* |
|  | *Neisseria flavescens* |
|  | *Neisseria macacae* |
|  | *Neisseria mucosa* |
|  | *Neisseria perflava* |
|  | *Neisseria sicca* |
|  | *Neisseria sp HMSC064E01* |
|  | *Neisseria sp oral taxon 014* |
|  | *Neisseria subflava* |
| PWY-7883: anhydromuropeptides recycling II | *Kingella denitrificans* |
|  | *Morococcus cerebrosus* |
|  | *Neisseria bacilliformis* |
|  | *Neisseria elongata* |
|  | *Neisseria macacae* |
|  | *Neisseria mucosa* |
|  | *Neisseria sicca* |
|  | *Neisseria sp HMSC064E01* |
|  | *Streptococcus pneumoniae* |
| PWY-6803: phosphatidylcholine acyl editing | *Campylobacter concisus* |
|  | *Campylobacter showae* |
|  | *Capnocytophaga sp oral taxon 338* |
|  | *Kingella denitrificans* |
|  | *Morococcus cerebrosus* |
|  | *Neisseria bacilliformis* |
|  | *Neisseria cinerea* |
|  | *Neisseria elongata* |
|  | *Neisseria flavescens* |
|  | *Neisseria macacae* |
|  | *Neisseria meningitidis* |
|  | *Neisseria mucosa* |
|  | *Neisseria perflava* |
|  | *Neisseria sicca* |
|  | *Neisseria sp HMSC064E01* |
|  | *Neisseria sp oral taxon 014* |
|  | *Neisseria subflava* |
|  | *Tannerella forsythia* |
| PWY-7200: superpathway of pyrimidine deoxyribonucleoside salvage | Unclassified |
| PWY-7323: superpathway of GDP-mannose-derived O-antigen building blocks biosynthesis | *Selenomonas sputigena* |
| PWY-7371: 1,4-dihydroxy-6-naphthoate biosynthesis II | *Campylobacter concisus* |
|  | *Campylobacter curvus* |
|  | *Campylobacter gracilis* |
|  | *Campylobacter rectus* |
|  | *Campylobacter showae* |
|  | *Centipeda periodontii* |
|  | *Desulfobulbus oralis* |
|  | *Mitsuokella sp oral taxon 131* |
|  | *Selenomonas artemidis* |
|  | *Selenomonas flueggei* |
|  | *Selenomonas infelix* |
|  | *Selenomonas noxia* |
|  | *Selenomonas sp F0473* |
|  | *Selenomonas sp FOBRC6* |
|  | *Selenomonas sp oral taxon 126* |
|  | *Selenomonas sp oral taxon 892* |
|  | *Selenomonas sp oral taxon 920* |
|  | *Selenomonas sputigena* |
| PWY-7456: &beta;-(1,4)-mannan degradation | Unclassified |
| PWY-7992: superpathway of menaquinol-8 biosynthesis III | *Campylobacter concisus* |
|  | *Campylobacter curvus* |

**
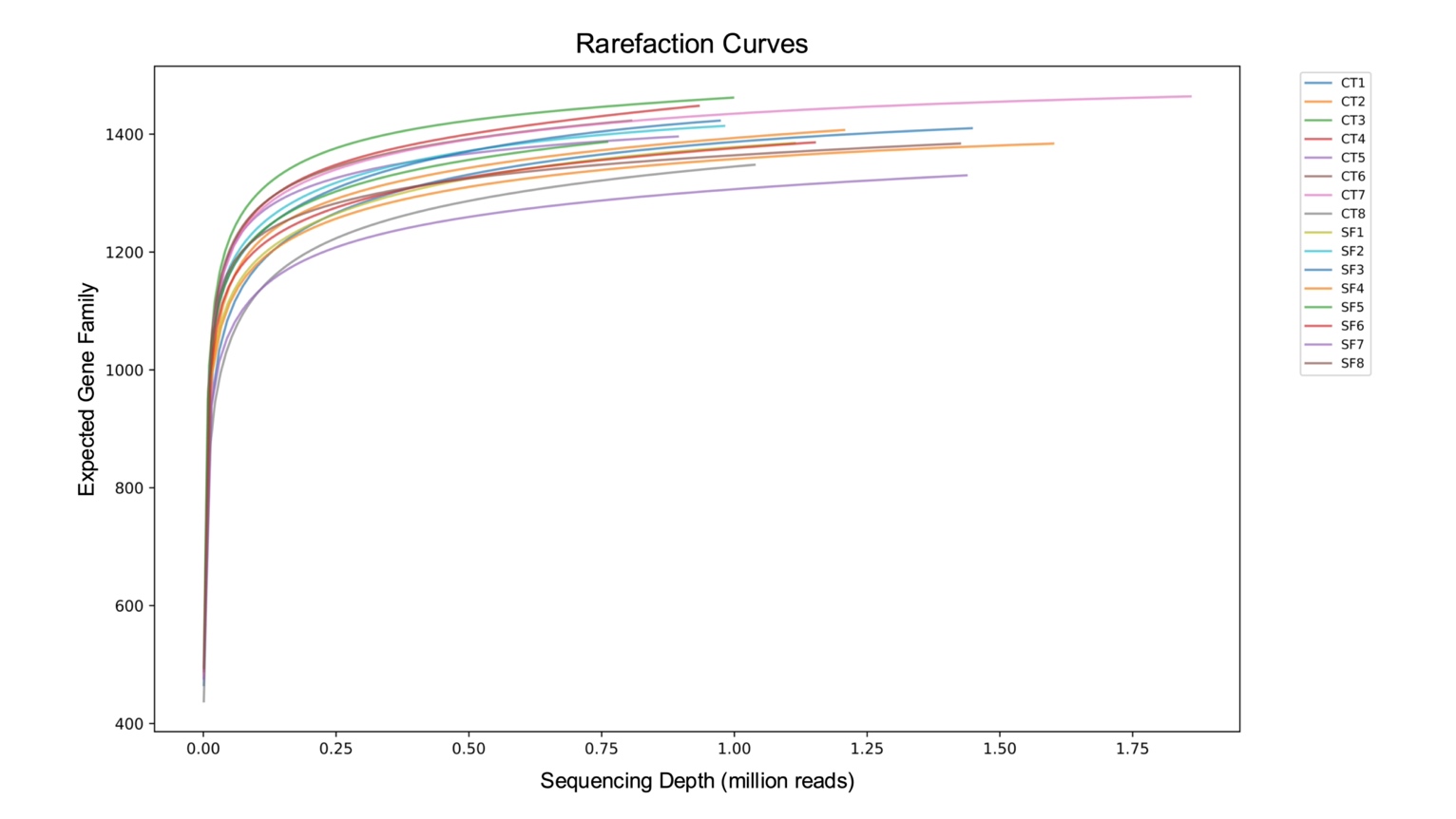
**

**Supplementary Figure S1. Rarefaction analysis of expected gene families and sequencing depth.** The plot shows the number of unique gene families observed (Expected Gene Family) as a function of increasing sequencing depth (million reads). Each line represents an individual sample from either the control (CT) or severe fluorosis (SF) group.

**
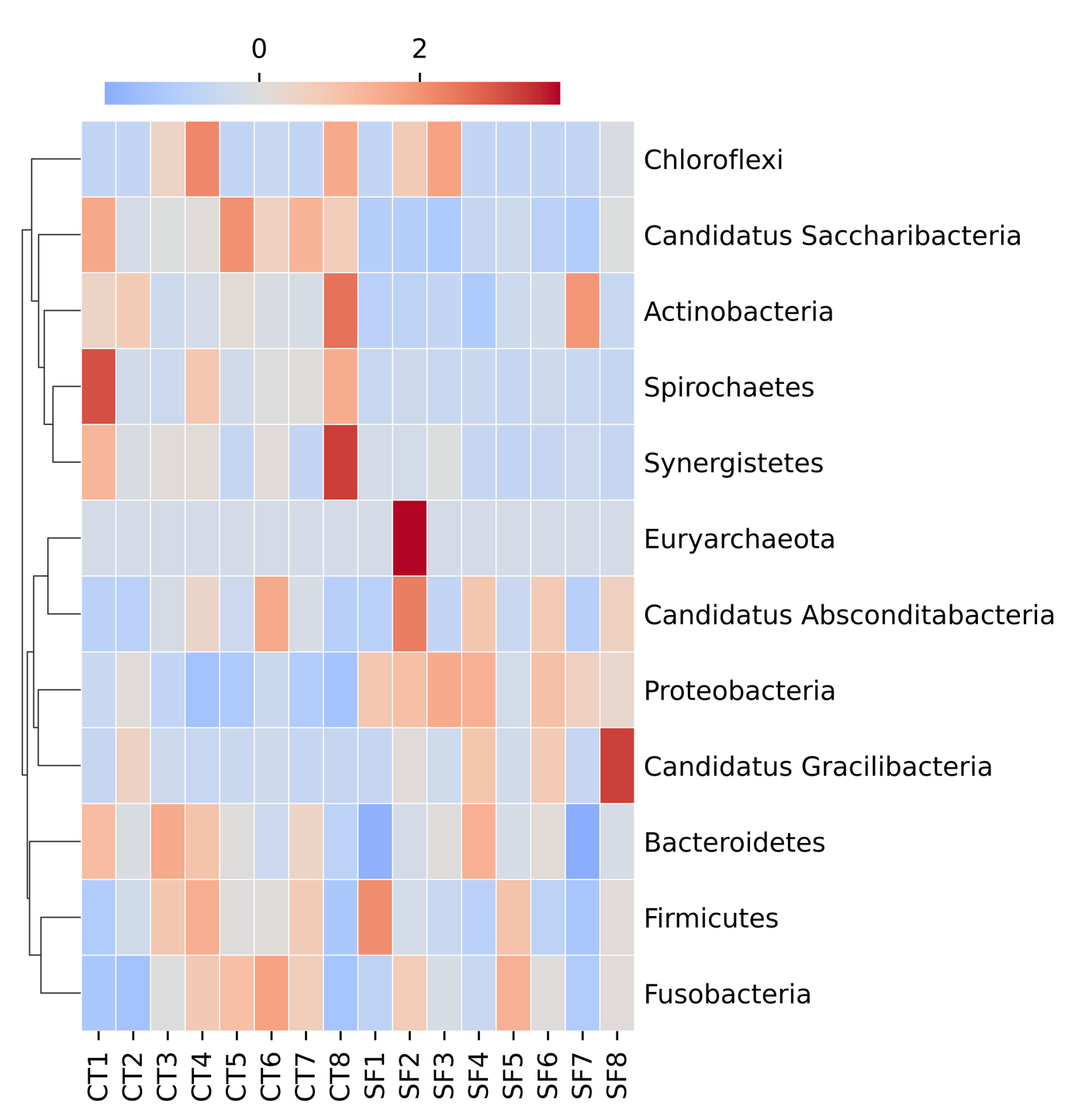
**

**Supplementary Figure S2. Heatmap of the 20 most abundant phyla across individual subjects.** The relative abundance data for each phylum was z-score normalized. The color key represents the z-score values. CT = control, SF = severe fluorosis.

**
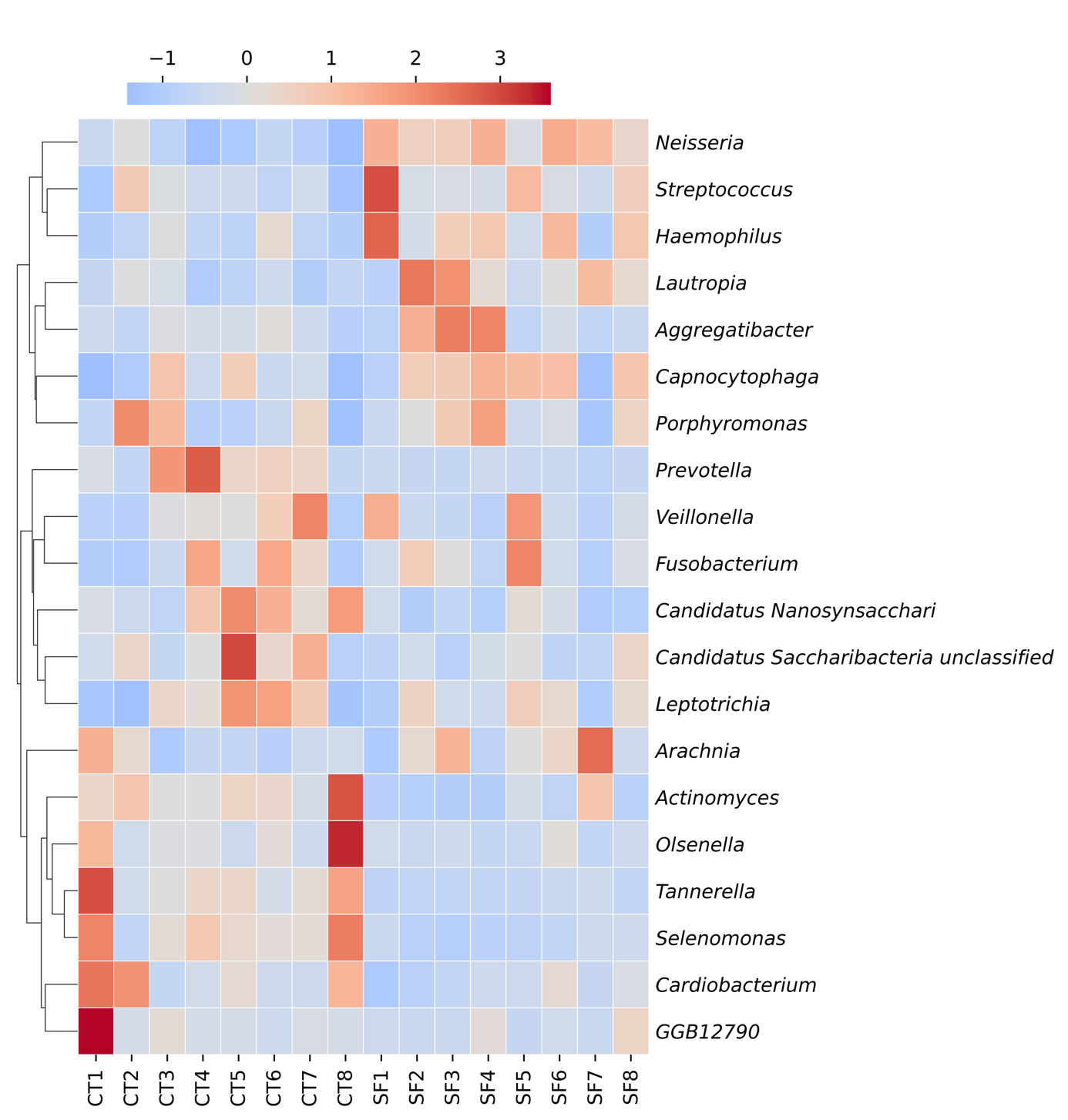
**

**Supplementary Figure S3. Heatmap of the 20 most abundant genera across individual subjects.** The relative abundance data for each genus was z-score normalized. The color key represents the z-score values. CT = control, SF = severe fluorosis.

**
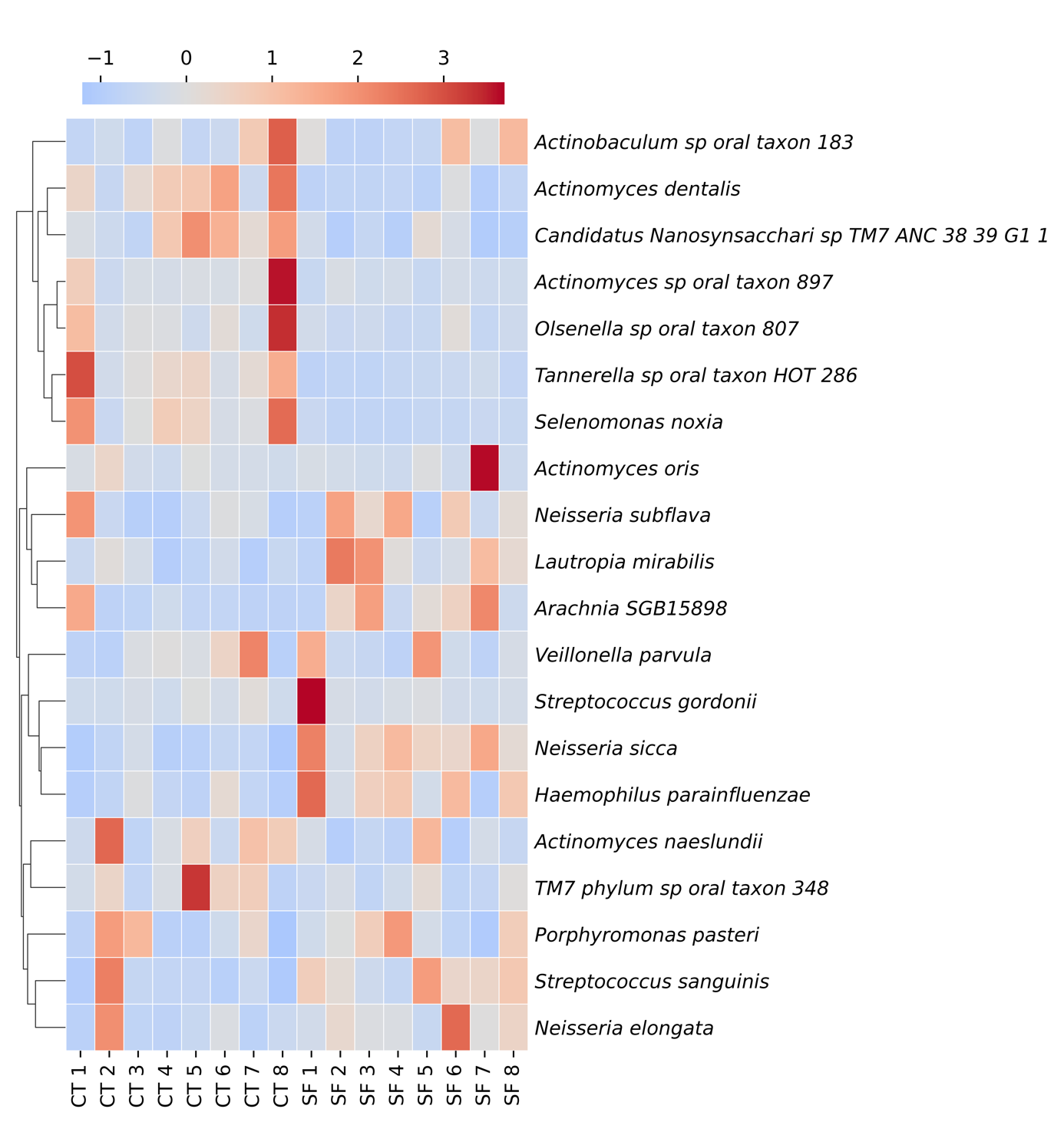
**

**Supplementary Figure S4. Heatmap of the 20 most abundant species across individual subjects.** The relative abundance data for each species was z-score normalized. The color key represents the z-score values. CT = control, SF = severe fluorosis.
